# Effect of Foliar Biostimulant Application on Bioactive Compounds and Antioxidant Capacity in Blueberry (*Vaccinium corymbosum* L.)

**DOI:** 10.3390/plants15010092

**Published:** 2025-12-27

**Authors:** Tiago Lopes, Ana Paula Silva, Alfredo Aires, Rosa Carvalho, Maria Ferreira, António A. Vicente, Berta Gonçalves

**Affiliations:** 1 Centre for the Research and Technology of Agro-Environmental and Biological Sciences (CITAB), University of Trás-os-Montes e Alto Douro (UTAD), 5000-801 Vila Real, Portugal; asilva@utad.pt (A.P.S.); alfredoa@utad.pt (A.A.); bertag@utad.pt (B.G.); 2 Institute for Innovation, Capacity Building and Sustainability of Agri-Food Production (Inov4Agro), University of Trás-os-Montes e Alto Douro (UTAD), 5000-801 Vila Real, Portugal; 3 Centre of Biological Engineering (CEB), University of Minho (UM), Campus de Gualtar, 4710-057 Braga, Portugal; avicente@deb.uminho.pt; 4 LABBELS—Associate Laboratory, 4710-057 Braga, Portugal; 5 Department of Agronomy, University of Trás-os-Montes e Alto Douro (UTAD), 5000-801 Vila Real, Portugal; rpaula@utad.pt (R.C.); al66431@alunos.utad.pt (M.F.); 6 Department of Biology and Environment, University of Trás-os-Montes e Alto Douro (UTAD), 5000-801 Vila Real, Portugal

**Keywords:** glycine betaine, *Ecklonia maxima*, phenolic compounds, *ortho*-diphenols, flavonoids, anthocyanins

## Abstract

Blueberries are rich in bioactive compounds and antioxidants whose contents can be significantly affected by pre-harvest agronomic practices. Thus, using natural biostimulants like *Ecklonia maxima* (EM) extract and glycine betaine (GB) is a promising strategy to improve blueberry quality. The effect of foliar pre-harvest application of EM and GB on bioactive compounds and antioxidant capacity (AC) of ‘Duke’ and ‘Draper’ blueberries was investigated in 2022 and 2023. Blueberries treated with GB had higher polyphenol contents and AC by the ABTS^•+^ and CUPRAC methods, particularly at higher doses. Vitamin C in ‘Duke’ was decreased by both doses of GB and low-dose EM + GB by 10–30% over two years, whereas in ‘Draper’, both doses of GB increased vitamin C in 2023 by 40–80%. High GB and EM + GB doses improved the AC of ‘Duke’ blueberries by 7–12% (CUPRAC) in both seasons. In 2022, the high dose of EM increased the levels of polyphenols in both cultivars by 14–21% and AC by ABTS^•+^ and CUPRAC in cv. ‘Draper’. As a result, blueberries’ nutritional value and AC can be enhanced by biostimulants, whose application must be adjusted according to cultivar and dose to optimize their use.

## 1. Introduction

Over the last decade, blueberries have become more popular mostly because of their excellent sensory attributes and beneficial health effects, including anti-inflammatory, antioxidant, neuroprotective, vision-enhancing, and anticancer properties, linked to their composition in bioactive compounds [1,2,3,4]. However, the qualitative parameters of blueberries depend on several factors, such as growing location, environmental conditions, cultivar, and fertilization strategy, among others [5,6,7,8].

Because of their complex chemical profile, blueberries are naturally rich in a variety of phenolic compounds, including flavonoids (e.g., anthocyanins, which are responsible for the characteristic pigmentation), *ortho*-diphenols, and other non-flavonoids, as well as vitamins and organic acids [3,4]. The antioxidant capacity (AC) of the fruit, which is an important factor determining its ability to scavenge free radicals, is mainly attributed to these compounds. Thus, elevated levels of these phytochemicals are directly responsible for the nutritional and functional improvements.

Regarding fertilization practices, the idea of using biostimulants to enhance tolerance to abiotic stresses, improve plant nutritional efficiency, and/or improve crop quality has been explored in recent decades across different plant species. Among these, biostimulants derived from seaweed extracts and glycine betaine (GB) have been used in several studies [9]. Previous work has reported that seaweed extracts may be useful for mitigating abiotic stresses, such as drought in blueberries [10]. Studies have also shown that seaweed-based biostimulants improved the total amount of phenolic compounds, anthocyanins, and antioxidant capacity (AC) of ‘Bluecrop’ blueberries [7]. Koort et al. [11] also reported an increase in the anthocyanin content of the ‘Northblue’ cultivar when treated with brown seaweed extracts, compared with mineral fertilization. Brown seaweeds, particularly from *Ecklonia maxima* (EM) species, contain several plant hormones, brassinosteroids, betaines, polyamines, phlorotannins, and alginate oligosaccharides, which can be responsible for the positive effects observed on bioactive compound contents and plant adaptation to environmental stresses [12]. On the other hand, GB, a non-toxic and highly water-soluble compound, has a fundamental role in osmotic adjustment while also protecting the photosynthetic apparatus, specifically photosystem II (PSII), from abiotic stress. GB stabilizes membrane integrity and various complex protein structures while preventing the accumulation of excess reactive oxygen species (ROS) [13]. The increase in yield and quality of different fruit crops was recently observed after the exogenous application of this compound [14,15,16]. In addition, the foliar application of GB has contributed to reducing enzyme activity crucial for energy metabolism and increasing the AC of ‘Brightwell’ blueberries, thus helping to maintain their post-harvest quality [17]. Recently, Lopes et al. [16] demonstrated that pre-harvest foliar application of EM and GB can enhance fruit size, firmness, and organic acid levels in blueberries of the ‘Duke’ and ‘Draper’ cultivars.

Although these positive effects have been observed on the physical and sensory attributes of blueberries, studies on the effects of foliar applications of EM- and GB-based biostimulants on phytochemical quality remain underexplored. Considering that the efficacy of biostimulants is influenced by cultivar, application method, and growing season, the aim of this research was to investigate whether the bioactive compound content and AC of blueberries would be significantly enhanced across different years by repeated foliar applications of different biostimulants, and whether the effects were synergistic, cultivar-dependent, and stable across different growing seasons. Therefore, the objective of this study was to evaluate the effect of pre-harvest foliar applications of EM- and GB-based biostimulants on the bioactive compounds and AC of ‘Duke’ and ‘Draper’ blueberry cultivars.

## 2. Results and Discussion

### 2.1. Total Phenolic Compounds, Flavonoids, and Ortho-Diphenols

**Figure 1:**
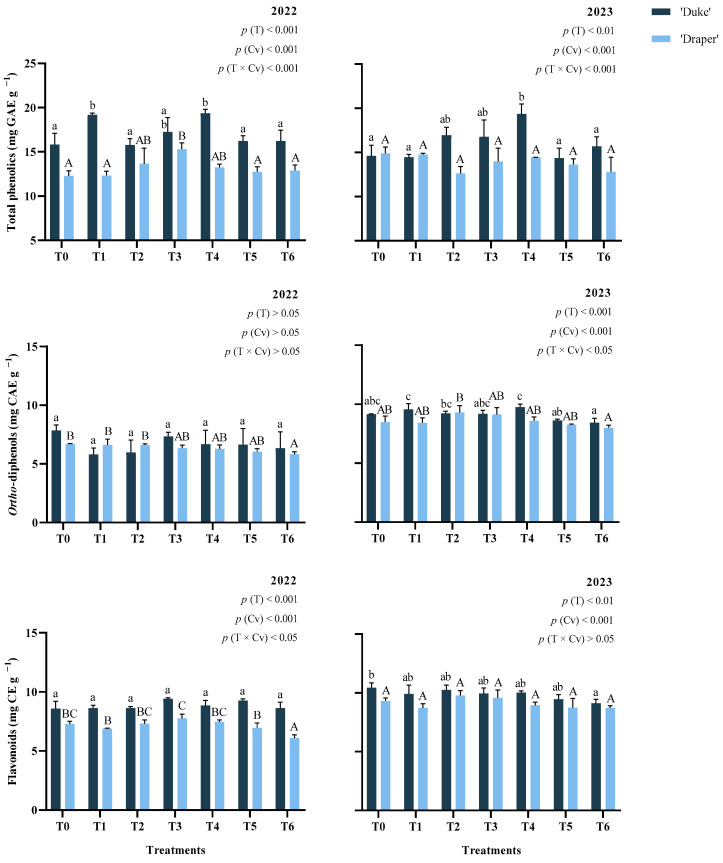
Total phenolics, flavonoids, and *ortho*-diphenols contents of fruits from ‘Duke’ and ‘Draper’ blueberry cultivars (Cv) depending on the treatments (T) used in the years 2022 and 2023. Lowercase letters indicate significant differences (*p* < 0.05) between the ‘Duke’ treatments and uppercase letters indicate significant differences (*p* < 0.05) between the ‘Draper’ treatments by Tukey’s test; means (*n* = 3) ± SD.

The contents of total phenolic compounds (TPC), flavonoids, and *ortho*-diphenols are shown in Figure 1. In both years, the foliar treatment (0.001 < *p* < 0.01), cultivar (*p* < 0.001), and the interaction treatment × cultivar (*p* < 0.001) significantly affected the TPC. GB-treated (T4) ‘Duke’ blueberries had significantly 22% higher TPC in 2022 (*p* < 0.001) and 33% in 2023 (*p* < 0.01) compared with the control. In 2022, 4 L ha^−1^ EM (T1) treatment significantly increased (*p* < 0.001) the TPC of ‘Duke’ fruits by 21%. For cv. ‘Draper’, the application of 4 kg ha^−1^ GB (T3) also increased (*p* < 0.01) TPC by 25%, compared with T0. Flavonoids were affected by treatment (*p* < 0.01) and cultivar (*p* < 0.001) in both years. However, the pattern was less consistent than for TPC, since the interaction treatment × cultivar was only significant in 2022 (*p* < 0.05). In that season, the use of EM + GB (T6) decreased (*p* < 0.001) the flavonoid content of ‘Draper’ blueberries by 16%, and in 2023, the same treatment also reduced (*p* < 0.05) flavonoids of ‘Duke’ fruits by 13%. This indicates a possible cultivar-specific sensitivity to combined treatments. The concentration of *ortho*-diphenols was affected by treatment (*p* < 0.001), cultivar (*p* < 0.001), and the interaction between these factors (*p* < 0.05) in the second year. For cv. ‘Draper’, the use of T6 also decreased the *ortho*-diphenol content of blueberries by 13% in 2022.

Interestingly, across both cultivars, flavonoid and *ortho*-diphenol contents were higher (*p* < 0.001) in 2023 than in the previous year, which can be explained by the higher average temperatures and solar radiation reported during the months of fruit development. The interaction between treatment, cultivar, and year was also significant for TPC (*p* < 0.001) and flavonoids (*p* < 0.05) (Table A1, Appendix A).

Previous research has shown that foliar spraying of biostimulants improves the levels of bioactive compounds in fruits, although the effect depends on cultivar and edaphoclimatic conditions. The increase in bioactive compounds after the use of biostimulants is consistent with studies in sweet cherries, grapes, and blueberries [10,14,15,18,19]. The higher concentration of phenolic compounds enhances plant tolerance against several biotic and abiotic stresses [20,21]. Indeed, these compounds can modulate gene expression related to the plant response to stress through the shikimate pathway, using phenylalanine and shikimic acid as precursors [22].

At the physiological level, the increase in TPC observed in the present work after GB treatment may be related to the stimulation of phenylpropanoid metabolism and the increase in associated enzyme activity [23]. Furthermore, GB can help prevent the oxidative degradation of phenolic compounds and the accumulation of ROS by improving enzymatic and non-enzymatic antioxidant systems [24]. As observed in this work, EM application can influence the levels of various phenolic compounds. These extracts contain several bioactive compounds, including phlorotannins, which can enhance phenolic synthesis by affecting key enzymes such as phenylalanine ammonia-lyase, peroxidase, and polyphenol oxidase [12,25]. In addition, an earlier study established that seaweed-based biostimulants can enhance the activity of genes involved in phenolic pathways that have revealed the potential to stimulate the biosynthesis of flavones [26].

Nonetheless, the accumulation of TPC and related compounds in treated plants can be affected by multiple factors. In a study on strawberries under water-stress conditions, Kapur et al. [27] found that some phenolic compounds were reduced while others increased following biostimulant treatment, reflecting the high complexity of the factors influencing these compounds. The increase in TPC observed in the present study is supported by previous findings on the effects of seaweed- and GB-based biostimulants in blueberries and other fruits like strawberries, sweet cherries, and grapes [7,15,18,19,28]. Post-harvest GB application has also improved TPC in blueberries [17]. On the other hand, decreased flavonoid and *ortho*-diphenol content in ‘Draper’ fruits treated with T6 in 2022 and in ‘Duke’ fruits in 2023 may suggest a cultivar-specific sensitivity or the stress-mitigating effect of the biostimulants. Based on this, although our results showed that foliar application of biostimulants generally enhanced TPC in blueberries, their effects on flavonoids and *ortho*-diphenols were more variable, which reflects a significant influence of cultivar, treatment, and environmental conditions.

### 2.2. Individual Phenolic Compounds Identified by HPLC

The individual polyphenol content, as determined by HPLC analysis, is presented in Table 1 and Table 2. Blueberries are rich in anthocyanins, which are known for their important role in preventing chronic diseases and several other beneficial health effects [29,30]. The main anthocyanins identified in our study were delphinidin-3-*O*-galactoside, delphinidin-3-*O*-glucoside, delphinidin-3-*O*-arabinoside, petunidin-3-*O*-galactoside, malvidin-3-*O*-galactoside, and malvidin-3-*O*-arabinoside, while chlorogenic acid (phenolic acid), rutin, and hyperoside (flavonols) were also detected. Consistent with our work, Yang et al. [31] recorded that, among 17 blueberry cultivars studied, more than 90% of identified anthocyanins were malvidin, delphinidin, and petunidin glycosides.

Results showed that both the sum of anthocyanins and the sum of individual polyphenols were affected by treatment (*p* < 0.01), cultivar (*p* < 0.001), and their interaction (0.001 < *p* < 0.01) during both years, as well as by the three-way interaction between treatment, cultivar, and year (*p* < 0.001) (Table A1 in Appendix A). Regarding pre-harvest foliar treatments, application of the low dose of EM (T2) decreased (*p* < 0.01) the sum of anthocyanins and individual polyphenols in ‘Duke’ blueberries by 20% and 17%, respectively, compared to the control (T0). In 2023, EM + GB treatments also decreased these compounds by 24% and 20%, respectively, for T5, and 28% and 24% for T6, when compared to T0. Conversely, EM (T1) and GB (T3) increased (*p* < 0.01) the sum of anthocyanins and the sum of individual polyphenols for cv. ‘Draper’ in 2022 by 21% and 29%, respectively.

The contrasting responses among cultivars regarding anthocyanin accumulation might be a result of environmental conditions and genotypic differences, since both abiotic and biotic stresses have been shown to influence anthocyanin biosynthesis and modulate the expression of key anthocyanins such as malvidin and delphinidin glycosides [31]. Consequently, the observed decrease in anthocyanin and total individual polyphenol contents following biostimulant application may indicate that, under low environmental stress, these treatments reduce the requirement for increased antioxidant production [22]. In addition, previous research has found a negative correlation between blueberry weight and anthocyanin concentration [5]. Thus, the increase in blueberry size observed in our recent study following the spraying of EM- and GB-based biostimulants application [16] might explain these results.

On the other hand, the increment of anthocyanin and individual polyphenol contents in ‘Draper’ fruits following the application of EM and GB in 2022 could also be attributed to a cultivar-specific response to these treatments. In line with our findings, previous work has shown that treatments with marine algae improve the content of delphinidin-3-O-galactoside and total polyphenols in ‘Bluecrop’ blueberries [7]. Similar effects were reported by Frioni et al. [32] on anthocyanins and phenolic contents of grapevine skin when a seaweed-based biostimulant was used. Such effects have been linked to the upregulation of genes responsible for anthocyanin transport by seaweed-based biostimulants, thereby supporting increases in anthocyanin content [26]. However, other investigations on seaweed-based biostimulant applications produced different results. An example of this is the study by Kapur et al. [27], which found that treatment with seaweed extract did not significantly alter the monomeric anthocyanin content of strawberries, suggesting that anthocyanin accumulation could be regulated by several factors. Regarding the external application of GB, transcriptome analysis suggests that this compound stimulates flavonoid biosynthesis by upregulating genes associated with flavonoid metabolic pathways [33]. In line with this, it has been shown that post-harvest application of GB led to an increase in anthocyanin content in ‘Brightwell’ blueberries [17], while similar effects were also observed in other crops, particularly in sweet cherries, in which the concentrations of cyanidin-3-*O*-rutinoside and cyanidin-3-*O*-glucoside increased after pre-harvest application of GB [34].

### 2.3. Vitamin C

**Figure 2:**
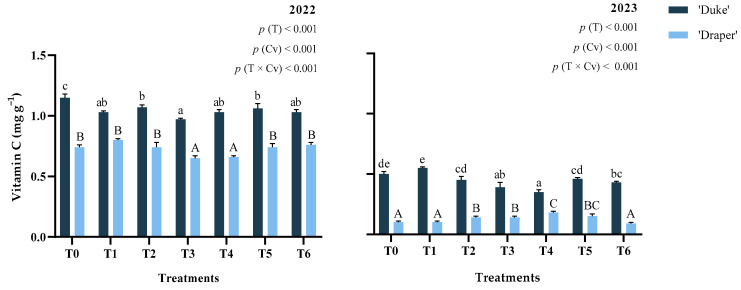
Vitamin C content of fruits from ‘Duke’ and ‘Draper’ blueberry cultivars (Cv) depending on the treatments (T) used in the years 2022 and 2023. Lowercase letters indicate significant differences (*p* < 0.05) between the ‘Duke’ treatments and uppercase letters indicate significant differences (*p* < 0.05) between the ‘Draper’ treatments by Tukey’s test; means (*n* = 3) ± SD.

Over the two experimental years, the average vitamin C content (Figure 2) for both cultivars ranged from 0.13 mg g^−1^ in cv. ‘Draper’ in 2023 to 1.05 mg g^−1^ in cv. ‘Duke’ in 2022. Similarly, a study conducted in Portugal by [6] with four highbush blueberry cultivars reported vitamin C levels between 0.06 and 1.7 mg g^−1^. Consistent with our results, Gündüz et al. [5] found higher vitamin C levels in ‘Duke’ blueberries compared to ‘Draper’ over three years.

Although vitamin C content is primarily determined by genotypic factors, climatic conditions, such as light intensity and water availability, can also influence its concentration [35]. These same authors claim that crops that are exposed to less frequent irrigation may contain more concentrated vitamin C. In our study, the higher temperatures and reduced precipitation during the stage of active fruit development in 2022, particularly in May, when solar radiation showed comparable levels in both years, may have contributed to an increase in vitamin C accumulation. This effect has been evidenced in other species, for example, in sweet cherries [36].

Vitamin C content was affected by treatment, cultivar, and their interaction (*p* < 0.001). This compound was also influenced by year and by the interaction treatment × cultivar × year (*p* < 0.001) (Table A1 in Appendix A). Results showed that ‘Duke’ fruits treated with GB (T3 and T4) presented lower vitamin C content in 2022 (16% and 10%, respectively) and in 2023 (22% and 30%, respectively), compared with T0. These treatments also reduced the vitamin C content by 12% and 11%, respectively, for ‘Draper’ blueberries. Additionally, lower (*p* < 0.001) vitamin C content was obtained in T6-treated ‘Duke’ blueberries, with reductions of 10% in 2022 and 14% in 2023. Similarly, in 2022, ‘Duke’ blueberries treated with EM (T1 and T2) and EM + GB (T5) showed a 10%, 7%, and 8%, respectively, lower (*p* < 0.001) concentration of this compound. Conversely, in 2023, the use of 2 L ha^−1^ EM (T2), both GB doses (T3 and T4) and 4 L ha^−1^ EM + 4 kg ha^−1^ GB (T5) increased *(p* < 0.001) the vitamin C concentration of ‘Draper’ blueberries by 40%, 40%, 80%, and 50%, respectively.

These contrasting effects suggest that, because vitamin C is produced as a plant response to stress, the application of GB-based biostimulants may be involved in plant internal defense mechanisms, resulting in the stabilization of proteins and cellular membranes, as well as in the interaction with stress-responsive genes. Therefore, GB may lower oxidative stress, thereby helping to neutralize reactive oxygen species (ROS), and the need for antioxidants like vitamin C [37,38]. Also, Gündüz et al. [5] found a negative correlation between fruit weight and its respective vitamin C concentration after studying several northern highbush cultivars over different years and locations, which suggests that the increased fruit weight and size following the application of biostimulants, as reported in our previous study [16], may have contributed to the decrease in vitamin C concentration.

Similar effects were reported by Correia et al. [36], who found a decrease in vitamin C in two cherry cultivars following foliar application of GB, compared with the untreated control. Regarding seaweed extract application, these authors observed different responses between cultivars, one of which showed increased vitamin C concentration, while in the other it decreased. Koort et al. [11] also observed a lower vitamin C content in ‘Northblue’ blueberries fertilized with organic fertilizer made from chicken manure and seaweed, as compared with mineral fertilization. Similarly, Ashour et al. [39] showed that vitamin C content was reduced in bell peppers after foliar application with seaweed extracts.

On the other hand, an increase in vitamin C content with the application of seaweed extract or GB through a foliar application method has been documented for several fruits [18,34,39,40,41]. In this context, the higher levels of vitamin C obtained after the application of these biostimulants might be linked to the improvement of plant health and metabolism, promoting the synthesis of various secondary metabolites, including bioactive compounds such as vitamin C. These results may indicate a potential genetic difference in response to vitamin C accumulation between the two blueberry cultivars, with genotype being a potential modifier of the response to biostimulants.

### 2.4. Antioxidant Capacity

The AC measured by the ABTS^•+^, CUPRAC, DPPH^•^, and FRAP methods can be observed in Table 3. In 2022, the ABTS^•+^, CUPRAC, and FRAP methods registered significant differences between treatments (*p* < 0.001), whereas in 2023, all AC methods were affected by treatments (0.001 < *p* < 0.01). Additionally, all AC parameters were statistically different among cultivars during both years (*p* < 0.001), confirming that genotype is an important factor that determines blueberry antioxidant activity [42]. Regarding the interaction treatment × cultivar, there was a statistically significant effect (0.001 < *p* < 0.01) in both AC methods for 2022. In the following year, the interaction was influenced only by the DPPH^•^ and FRAP methods (*p* < 0.001). All AC assays were also affected (*p* < 0.001) by the interaction treatment × cultivar × year (Table A1 in Appendix A).

The average AC values determined by the CUPRAC and FRAP assays were significantly higher (*p* < 0.001) in 2022, whereas the ABTS^•+^ and DPPH^•^ assays had higher (*p* < 0.001) values in 2023. In 2022, the application of EM + GB (T5 and T6) showed a 9% and 8% increase in the CUPRAC method, respectively, compared with T0. In addition, FRAP values of ‘Duke’ blueberries recorded 9% and 7% increases, respectively, after the application of T5 and T6. For cv. ‘Draper’, the treatment with 4 L ha^−1^ EM (T1), both doses of GB (T3 and T4), and 4 L ha^−1^ EM + 4 kg ha^−1^ GB (T5) showed higher (*p* < 0.001) AC as determined by the ABTS^•+^ assay by 17%, 29%, 37%, and 25%, respectively, and by the CUPRAC by 14%, 21%, 12%, and 14%, respectively.

**Figure A1:**
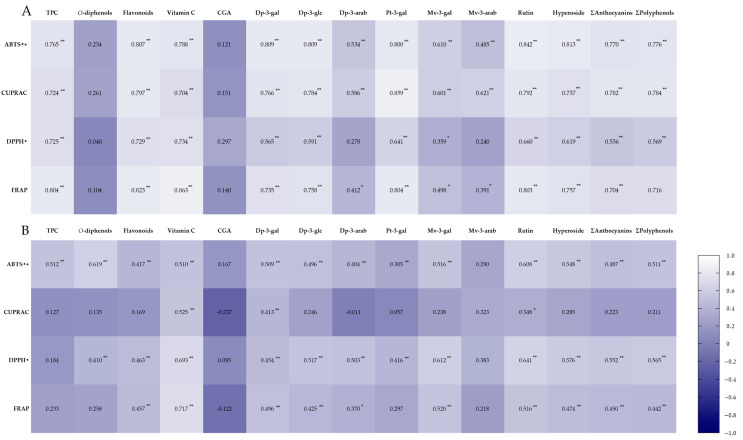
Pearson’s correlation analysis between bioactive compounds and AC across the different methods of blueberries from both cultivars in the years 2022 (**A**) and 2023 (**B**). Abbreviations: TPC—total phenolic compounds; O-diphenols—*ortho*-diphenols; CGA—chlorogenic acid; Dp-3-gal—delphinidin-3-*O*-galactoside; Dp-3-glc—delphinidin-3-*O*-glucoside; Dp-3-arab—delphinidin-3-*O*-arabinoside; Pt-3-gal—petunidin-3-*O*-galactoside; Mv-3-gal—malvidin-3-*O*-galactoside; Mv-3-arab—malvidin-3-*O*-arabinoside; ΣAnthocyanins—sum of anthocyanins; ΣPolyphenols—sum of individual polyphenols. * means correlation is significant at *p* < 0.05; ** correlation is significant at *p* < 0.01.

**Figure A2:**
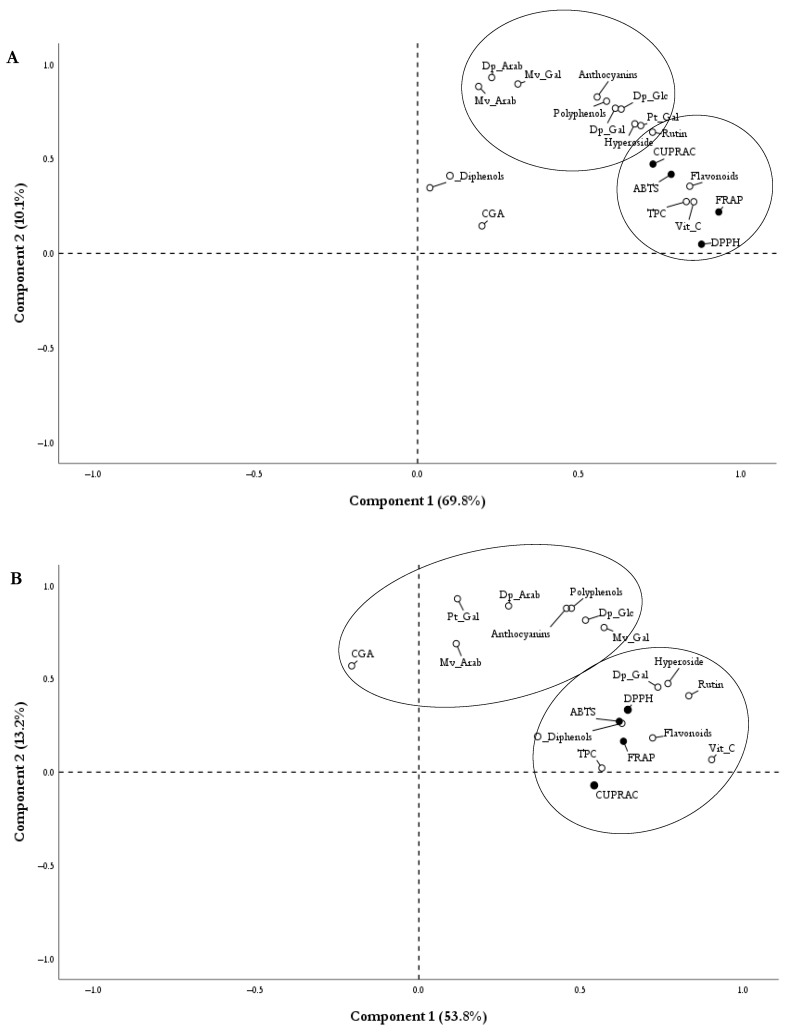
Principal component analysis (PCA) of bioactive compounds and AC of blueberries from both cultivars in the years 2022 (**A**) and 2023 (**B**). Ellipses indicate variable associations based on the loadings of PC1 and PC2. Abbreviations: TPC—total phenolic compounds; O_Diphenols—*ortho*-diphenols; Vit_C—vitamin C; CGA—chlorogenic acid; Dp_Gal—delphinidin-3-*O*-galactoside; Dp_Glc—delphinidin-3-*O*-glucoside; Dp_Arab—delphinidin-3-*O*-arabinoside; Pt_Gal—petunidin-3-*O*-galactoside; Mv_Gal—malvidin-3-*O*-galactoside; Mv_Arab—malvidin-3-*O*-arabinoside; Anthocyanins—sum of anthocyanins; Polyphenols—sum of individual polyphenols.

Taken together, these results might be attributed to the concentration of bioactive compounds in treated plants, which correlate closely with the AC measured by the methods assayed, as revealed by the results of Pearson’s correlation analysis (Figure A1 in Appendix A) and principal component analysis (PCA) (Figure A2 in Appendix A). The PCA, which incorporated all antioxidant and phenolic parameters for each year, explained 79.9% and 67.0% of total variance in 2022 and 2023, respectively. Thus, in both years, PC1 was closely related to TPC, flavonoids, rutin, vitamin C, and to all antioxidant capacity assays (CUPRAC, FRAP, ABTS^•+^, and DPPH^•^), illustrating the general antioxidant potential of the fruit. Also, PC2 was associated with the individual phenolic compounds, particularly anthocyanin derivatives, reflecting differences in specific phenolic composition across the samples.

Regarding cv. ‘Draper’, the results of T1 and T3 application on AC can be explained by the positive correlation (*p* < 0.001) of total anthocyanins and total individual polyphenols with CUPRAC (*r* = 0.782 and *r* = 0.784, respectively) and ABTS^•+^ (*r* = 0.770 and *r* = 0.776, respectively) (Figure A1 in Appendix A). T3 sprays increased TPC, which, in turn, showed a positive correlation (*p* < 0.001) with ABTS^•+^ (*r* = 0.765) and CUPRAC (*r* = 0.724). Likewise, we observed that T4 increased rutin content, which correlated positively (*p* < 0.001) with the ABTS^•+^ (*r* = 0.842) and CUPRAC (*r* = 0.792) methods. Additionally, T5-treated fruits had higher delphinidin-3-*O*-galactoside and delphinidin-3-*O*-arabinoside contents, which had a positive correlation (*p* < 0.001) with CUPRAC (*r* = 0.766 and *r* = 0.586, respectively) and ABTS^•+^ (*r* = 0.809 and *r* = 0.534, respectively).

In contrast, T2 decreased (*p* < 0.001) the CUPRAC values in cv. ‘Duke’ by 16% in 2022, while this treatment increased (*p* < 0.001) the AC values in ‘Draper’ fruits by 14%. DPPH^•^ results indicate that the high dose of EM + GB (*p* < 0.01) decreased by 22% the AC of ‘Draper’ blueberries as compared with T0. For cv. ‘Duke’, the decrease in AC measured by the CUPRAC method following the application of T2 might be related to the reduction in the sum of anthocyanins, the sum of individual polyphenols, and vitamin C, which showed strong positive correlations with this method (*r* = 0.782, *r* = 0.784, and *r* = 0.704, respectively; *p* < 0.001). In both years, results showed that the application of 4 kg ha^−1^ GB (T3) increased (*p* < 0.001) the AC of ‘Duke’ blueberries according to the CUPRAC method by 7% in 2022 and 12% in 2023. In 2023, regarding cv. ‘Duke’, the application of the high dose of EM (T1) increased (*p* < 0.001) the AC by 11% according to the ABTS^•+^ method and by 24% according to the DPPH^•^. Similarly, ‘Duke’ blueberries treated with T5 showed 11% higher (*p* < 0.001) CUPRAC values, with a similar effect (*p* < 0.01) regarding FRAP results (15% increase) in the cultivar ‘Draper’. Exogenous T3 application increased AC levels as measured by the CUPRAC method in both years. This increase might be explained by the higher concentrations of malvidin-3-*O*-arabinoside in 2023, which also showed a positive correlation (*r* = 0.323; *p* < 0.05) with this assay. In the case of ‘Draper’ blueberries treated with T5, the increase in AC measured by the FRAP can be attributed to the higher vitamin C concentration, positively correlated with this method (*r* = 0.717; *p* < 0.001).

Seaweed-based biostimulants can improve fruit development and quality by enhancing its antioxidant properties [43]. According to the literature, the efficiency of EM in increasing AC might be related to its composition in bioactive compounds, affecting plant metabolism and promoting the activity of several antioxidant enzymes that lower oxidative stress [43,44,45]. On the other hand, GB also controls the production of antioxidant enzymes, such as ascorbate peroxidase, catalase, peroxidase, and superoxide dismutase, in crops [24,26]. As a consequence, this can lead to higher AC levels, as observed in the present study. Secondly, GB could also improve antioxidant systems indirectly by the improvement of sugar and organic acid content [46]. Overall, these biostimulants may enhance the phenylpropanoid pathway and improve the activity of antioxidant enzymes, enabling plants to better tolerate several environmental stresses [47].

Regarding the pre-harvest application of a marine algae-based biostimulant, Lenart et al. [7] found higher levels of AC in ‘Bluecrop’ blueberries assessed by the DPPH^•^ method. According to this assay, post-harvest application of GB in ‘Brightwell’ blueberries also resulted in higher AC values compared with the control group [17]. The authors attribute these findings to increased enzymatic and non-enzymatic antioxidants. Similar results were observed following the pre-harvest foliar application of GB, as determined by the FRAP method in grapes [26] and by the DPPH^•^ method in tomatoes [40] and bell peppers [39]. Similar effects were found for the application of a foliar seaweed-based biostimulant in cherries, where the treatment increased AC (DPPH^•^ and FRAP) compared with the control [19]. Furthermore, it has been shown that seaweed- and yeast-based biostimulants enhanced the antioxidant properties of tomatoes, measured by the DPPH^•^ and ABTS^•+^ assays [48].

However, a reduction in the AC was also observed in our study, depending on the antioxidant test used, following biostimulant application to blueberries. In 2023, ‘Duke’ blueberries treated with 2 kg ha^−1^ GB (T4) had 13% and 9%, respectively, lower (*p* < 0.001) CUPRAC and FRAP values compared with T0. Lower vitamin C content was observed in treated blueberries, which positively correlated with CUPRAC (*r* = 0.525; *p* < 0.001) and FRAP (*r* = 0.717; *p* < 0.001) assays. Fruits sprayed with T4 also showed lower delphinidin-3-*O*-galactoside, which correlated with CUPRAC (*r* = 0.413; *p* < 0.01) as well as FRAP (*r* = 0.496; *p* < 0.001). Both doses of EM + GB (T5 and T6) decreased (*p* < 0.001) FRAP values of ‘Duke’ blueberries in 2023 by 6% and 8%, respectively. Based on our results, it was possible to observe that T5 and T6 both reduced the levels of compounds correlated with the FRAP method, such as the sum of anthocyanins (*r* = 0.450; *p* < 0.01) and the sum of polyphenols (*r* = 0.442; *p* < 0.01), while T6 also reduced concentrations of vitamin C (*r* = 0.717; *p* < 0.001) and flavonoids (*r* = 0.457; *p* < 0.01).

Based on our results, foliar applications of EM and GB are therefore effective tools for improving the nutraceutical quality of blueberries. However, their effects are influenced by the interaction of genotype and environmental conditions. Thus, future research should focus on elucidating the mechanisms underlying these responses by quantifying the activity of antioxidant enzymes and enzymes of the phenylpropanoid pathway, as well as by expressing key genes responsible for flavonoid biosynthesis. Such investigations will clarify whether the observed increases in bioactive compounds and AC result from the transcriptional activation of these metabolic pathways, and hence the establishment of a direct link between biostimulant application, molecular responses, and improved fruit quality.

## 3. Materials and Methods

### 3.1. Plant Material and Sampling

Experiments were carried out in a commercial orchard located in Vilarandelo, Valpaços municipality (41°40′8.38″ N, 7°19′22.81″ W, 593 m asl), north of Portugal, for two years (2022 and 2023). ‘Duke’ and ‘Draper’ (*Vaccinium corymbosum* L.) northern highbush blueberry cultivars, planted in 2012, were selected due to their commercial importance and widespread cultivation in Portugal. Plants were spaced 3 × 1 m apart in a north–south orientation and irrigated using a drip irrigation system with two lines per ridge. The bushes were treated with the same fertilization program based on soil analysis during the trial. The soil contained 4.71% organic matter, moderate acidity (pH 5.6), medium texture, high phosphorus content (105 mg P_2_O_5_ kg^−1^), and very high potassium content (426 mg K_2_O kg^−1^).

*Ecklonia maxima* (EM) macroalgae (Kelpak^®^, Daymsa, Zaragoza, Spain) and glycine betaine (GB) (Greenstim^®^, Massó Agro Department, Barcelona, Spain) were used. Kelpak^®^ is a natural concentrate of 100% EM algae with a pH of 4.4, 20 mS/cm conductivity, and 0.55% K_2_O content (*w*/*w*), while Greenstim^®^ is made up of 97% GB obtained from sugar beet with a C/N ratio of 4.9, 56% organic C, 11.5% organic N, and 12% total N (*w*/*w*). A manual backpack sprayer equipped with a single adjustable nozzle was used for the application of biostimulants on groups of nine plants per treatment for each cultivar at three phenological stages: full bloom (BBCH 65), early green fruit (BBCH 71), and fruit coloring (BBCH 81) [49]. At each growth stage, the following treatments were applied: 4 L ha^−1^ (T1) and 2 L ha^−1^ (T2) of EM-based biostimulant; 4 kg ha^−1^ (T3) and 2 kg ha^−1^ (T4) of GB-based biostimulant; a combination of 4 L ha^−1^ EM + 4 kg ha^−1^ GB (T5) and 2 L ha^−1^ EM + 2 kg ha^−1^ GB (T6); and water as a control treatment (T0). The dosages for GB and EM were applied in accordance with the manufacturer’s recommendations. Applications were made in the morning, the canopy was fully covered, and no precipitation event was forecasted for the next 24 h.

‘Duke’ blueberries were picked at commercial maturity, according to cultivar traits, on 14 June (2022) and 12 June (2023), whereas ‘Draper’ blueberries were picked on 27 June (2022) and 20 June (2023). For every treatment, hundreds of fruits with uniform size were randomly collected and taken to the laboratory for analysis. The fruits were frozen in liquid nitrogen and stored at −80 °C until quantification of bioactive compounds and AC assays. Prior to analysis, blueberries were freeze-dried, ground into a powder using a commercial blender, and properly identified before being used in the analytical procedure. All determinations for chemical and biochemical analyses were performed in triplicate.

### 3.2. Climatic Conditions

**Figure 3:**
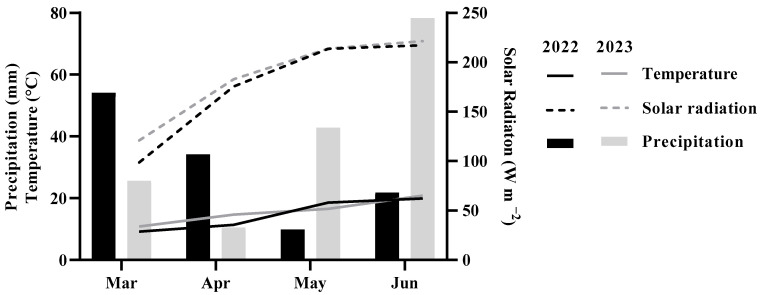
Mean temperature (°C), monthly precipitation (mm), and mean solar radiation (W m^−2^) for Vilarandelo in the period from March to June in 2022 and 2023.

The plantation area has a temperate climate according to the Köppen classification, with wet winters and dry, hot summers, classified as “Csa.” Temperature and precipitation data (Figure 3) were obtained from reanalysis datasets based on nearby meteorological stations, specifically the E-OBS dataset, whilst mean solar radiation was provided by the EMA network property of DRAPN whose data are controlled through mySense Platform. The average air temperature throughout the growing season, from March to June, was approximately 1.0 °C lower in 2022 compared to 2023. Nevertheless, a temperature about 2.0 °C higher was observed in May during the first season. Compared to the long-term air temperature of Vila Real (1981–2010), March and April 2022 were below average, while May and June exceeded the long-term mean. In contrast, all months in 2023 recorded temperatures above the climatological normals. The values of precipitation during March (54.1 mm) and April (34.2 mm) were higher in 2022, whereas in 2023 higher values were presented during May (42.8 mm) and June (78.3 mm). Overall, both years showed lower precipitation levels than the long-term, except for June 2023. In addition, the mean solar radiation was higher in 2023 than in 2022.

### 3.3. Determination of Phenolic Compounds

Prior to the analysis of TPC, flavonoids, *ortho*-diphenols, and antioxidant capacity (AC), a blueberry extract was prepared. For this, 40 mg dry weight (DW) of each sample was mixed with 1 mL of 70% methanol (*v*/*v*) in 2 mL centrifuge tubes. The mixture was homogenized using a vortex and then heated at 70 °C for 30 min. Afterwards, the samples were centrifuged at 11,000 rpm and 1 °C for 15 min (Centrifuge 5804R, Eppendorf, Hamburg, Germany). The supernatant was then collected and filtered with 0.2 µm Spartan filters into amber vials.

#### 3.3.1. Total Phenolic Compounds (TPC)

TPC were analyzed via the colorimetric Folin–Ciocalteu method of Singleton and Rossi [50] and Dewanto et al. [51], in a 96-well microplate. A total of 20 μL extract was mixed with 100 μL diluted Folin–Ciocalteu reagent (1:10 in bidistilled water) and 80 μL of 7.5% Na_2_CO_3_. The microplates were subsequently incubated in the dark at 45 °C for 15 min. Absorbance was measured at 765 nm using a microplate reader (Multiskan GO Microplate Spectrophotometer, Thermo Scientific, Vantaa, Finland). Results were expressed as milligrams of gallic acid equivalents per gram (mg GAE g^−1^), on a DW basis.

#### 3.3.2. *Ortho*-Diphenols

The determination of *ortho*-diphenols was made according to the methodologies described by Gutfinger [52] and Garcia et al. [53]. In each well of a 96-well microplate, 20 µL of sample was added, followed by the addition of 100 μL ultrapure water, 80 μL of phosphate buffer pH 6.5 (0.1 mol L^−1^), and 160 μL of 5% sodium molybdate solution Na_2_MoO_4_·2H_2_O. Then, the microplate was incubated in the dark at room temperature for 15 min. A calibration curve was prepared using caffeic acid (C_9_H_8_O_4_) as standard and absorbance values were recorded at 350 nm with a microplate reader (Multiskan GO Microplate Spectrophotometer, Thermo Scientific, Vantaa, Finland). Results were expressed in milligrams of caffeic acid equivalents per gram (mg CAE g^−1^), on a DW basis.

#### 3.3.3. Flavonoids

The determination of flavonoid content was performed using the colorimetric method adopted by Dewanto et al. [51], using 96-well microplates. In each well of the microplate, 25 µL of extract was mixed with 100 µL of ultrapure water and 10 µL of a 5% sodium nitrite (NaNO_2_) solution. The solution was then homogenized and incubated at room temperature in the dark for 5 min. Then, 15 µL of aluminum chloride (AlCl_3_) 10% was added and the microplate was placed again for 6 min at room temperature in the dark. Sodium hydroxide (NaOH) 1 mol L^−1^ (50 µL) and ultrapure water (50 µL) were added to each well. A calibration curve was prepared using catechin as standard and absorbances were recorded at 510 nm using a microplate reader (Multiskan GO Microplate Spectrophotometer, Thermo Scientific, Vantaa, Finland). The results were reported as milligrams of catechin equivalents per gram (mg CE g^−1^), on a dry weight (DW) basis.

### 3.4. Individual Polyphenols

Individual polyphenols were determined via HPLC-DAD-UV/VIS according to Aires et al. [54]. First, 40 mg of dried powder of blueberry sample was extracted using 950 µL of 70% methanol (*v*/*v*) and 50 µL of naringin (1 mg mL^−1^) as internal standard. The mixture was subjected to a warm bath at 70 °C for 30 min with intermittent stirring every 5 min and extracts were centrifuged at 11,000 rpm, 4 °C during 15 min (Centrifuge 5804R, Eppendorf, Hamburg, Germany). The supernatant was filtered using 0.20 µm cellulose ester filters (WhatmanTM, Spartan 13/0.2 RC, Maidstone, UK) and transferred to amber HPLC vials to prevent light degradation. After this process, vials were stored at −20 °C until analysis. The HPLC-DAD-UV/VIS analysis was carried out using a reverse-phase column (C18 Spherisorb ODS2, 250 mm × 4.6 mm), and eluents of 0.1% trifluoroacetic acid (TFA) in water (solvent A) and 0.1% TFA in acetonitrile (solvent B), in a run of 60 min, starting with 100% solvent A, while the flow rate was set to 1 mL min^−1^, and an injection volume of 10 µL. Chromatograms were recorded at 280, 320, and 370 nm for phenolic compounds and at 520 nm for anthocyanins, and identification was based on retention time, UV spectra of each compound, and comparison with commercial external standards. Quantification was performed using external calibration curves, internal standards, and the response factor for each detected polyphenol. The chromatographic profile of polyphenols is presented in Supplementary Figure S1. Results for individual polyphenols are expressed in µg g^−1^, whereas the sums of individual polyphenols and anthocyanins are expressed in mg g^−1^, on a DW basis.

### 3.5. Determination of Vitamin C Content

The vitamin C content was quantified using HPLC-DADUV/VIS according to Hernandez et al. [55], adapted by Aires et al. [56]. For the extraction, 200 mg of lyophilized blueberry sample was weighed and mixed with 5.0 mL extraction solvent comprising 3.0% metaphosphoric acid and 8% acetic acid as well as 1.0 mmol L^−1^ tert-butylhydroquinone (TBHQ) (Sigma-Aldrich, Tauferkichen, Germany). The samples were homogenized using a disperser (T 25 digital ULTRA-TURRAX^®^, Staufen, Germany) and then centrifuged at 4000 rpm for 5 min at 4 °C (Centrico 250, UniEquip, Munich, Germany). The supernatant was then filtered through PTFE 0.2 µm filters (Whatman™ Spartan 13/0.2 RC, Maidstone, UK) into amber HPLC vials and injected immediately. The analysis was conducted in an HPLC system equipped with a C18 reverse-phase column (Spherisorb ODS2, 250 × 4.6 mm, 5 μm) applying a mobile phase composed of 0.2% *ortho*-phosphoric acid in an isocratic gradient. The flow rate was set to 1.2 mL min^−1^. The injection volume was 20 µL, and the recording of chromatograms was performed at 245 nm. The identity of vitamin C was ascertained by the retention time of chromatographic peaks by comparing the same with an external standard of vitamin C (Sigma-Aldrich, Tauferkichen, Germany). Finally, the contents were presented in mg g^−1^, on a DW basis.

### 3.6. Antioxidant Capacity Assays

#### 3.6.1. ABTS^•+^ Radical-Scavenging Activity

ABTS^•+^ (2,2’-azino-bis (3-ethylbenzothiazoline-6-sulphonic acid)) discoloration assay was performed using the method of Stratil et al. [57] with modifications. A working solution was made by mixing 1 mg mL^−1^ ABTS^•+^ with 0.68 mg mL^−1^ potassium persulfate (K_2_S_2_O_8_) in distilled water. It was incubated at room temperature in the dark for 12 to 16 h. After this period, the solution was diluted with absolute ethanol to reach an absorbance between 0.8 and 1.0 at 734 nm. For quantification, 15 µL of the extract previously prepared was added in each well of the 96-well microplate, considering that the blank was made with 70% methanol (*v*/*v*). Then, 285 µL of ABTS^•+^ working solution was added, and the microplate was incubated in the dark for 10 min. A calibration curve was prepared using Trolox (Sigma-Aldrich, Tauferkichen, Germany) as standard and absorbances were recorded at 734 nm using a microplate reader (Multiskan GO Microplate Spectrophotometer, Thermo Scientific, Vantaa, Finland). Results were presented as micromoles Trolox equivalent (TE) per gram (µmol TE g^−1^, on a DW basis).

#### 3.6.2. Cupric-Reducing Antioxidant Capacity (CUPRAC)

The CUPRAC assay was made according to the method described by Apak et al. [58]. For the determination, solutions of 10 mmol L^−1^ CuCl_2_ in water, 7.4 mmol L^−1^ neocuproine (Sigma-Aldrich, Tauferkichen, Germany) in 96% ethanol, and 1 mmol L^−1^ ammonium acetate buffer solution at pH 7.0 in water were prepared. Then, 50 µL of CuCl_2_ solution, 50 µL of neocuproine solution, 50 µL of buffer solution, 25 µL of sample, and 25 µL of bidistilled H_2_O were added sequentially to each well of the 96-well microplate. The blank was composed by adding all reagents except CuCl2. The microplate was left in the dark at room temperature for 30 min and the absorbance was then measured at 450 nm using a microplate reader (Multiskan GO Microplate Spectrophotometer, Thermo Scientific, Vantaa, Finland). A calibration curve was performed with Trolox (Sigma-Aldrich, Tauferkichen, Germany) as standard at different concentrations. The results were expressed as μmol L^−1^ Trolox equivalents (TE) per gram of sample (μmol L^−1^ TE g^−1^), on a dry weight (DW) basis.

#### 3.6.3. DPPH^•^ Radical-Scavenging Capacity

DPPH^•^ radical scavenging capacity was assessed following the method of Siddhraju and Becker [59] adapted to 96-well microplates. An amount of 285 µL of DPPH^•^ solution prepared by dissolving 4 mg of 2,2-diphenyl-1-picrylhydrazyl radical in 100 mL of 95% ethanol was added to each microplate well, to which 15 µL of extract was added. A blank sample was prepared by adding all reagents except the extract, which was replaced with the solvent used for extraction. The microplate was left in the dark at room temperature for 30 min. A calibration curve was performed using Trolox (Sigma-Aldrich, Tauferkichen, Germany) as standard at different concentrations, and absorbances were recorded at 517 nm in a microplate reader (Multiskan GO Microplate Spectrophotometer, Thermo Scientific, Vantaa, Finland). Values were expressed as μmol L^−1^ Trolox equivalents per gram (μmol L^−1^ TE g^−1^), on a DW basis.

#### 3.6.4. Ferric-Reducing Antioxidant Power (FRAP)

The FRAP assay was performed according to Stratil et al. [57]. Initially, FRAP reagent was prepared by combining, in a ratio of 10:1:1 (*v*/*v*/*v*), 300 mmol L^−1^ acetate buffer at pH 3.6, 10 mmol L^−1^ TPTZ (2,4,6-tri(2-pyridyl)-s-triazine) in 40 mmol L^−1^ HCl, and 20 mmol L^−1^ FeCl_3_·6H_2_O. A calibration curve was also prepared using a solution of FeSO_4_ as standard at different concentrations. To each well of the microplate, 25 µL of sample or standard was added, followed by 275 µL of the FRAP reagent. The extraction solvent (70% methanol) was the blank. The microplates were therefore incubated in the dark at room temperature for 5 min. Then, the absorbance values were recorded at 593 nm in a microplate reader (Multiskan GO Microplate Spectrophotometer, Thermo Scientific, Vantaa, Finland). Results were expressed as µmol of FeSO_4_ equivalents per gram (µmol FeSO_4_ g^−1^), on a DW basis.

### 3.7. Statistical Analysis

Data were subjected to analysis of variance (ANOVA), followed by Tukey’s HSD post hoc test with a significance level of 5% for mean comparison. All the above tests were carried out using SPSS software version 27.0 (SPSS-IBM, Orchard Road, Armonk, New York, NY, USA). One-way, two-way, and three-way ANOVA were applied to compare the treatment and cultivar effect in a particular year, as well as the year effect. Pearson’s correlation test and a principal component analysis (PCA) were also performed to evaluate the relationships between bioactive compounds and AC.

## 4. Conclusions

This two-year study reveals that the foliar application of biostimulants, particularly those based on EM and GB, strongly affects the phenolic compounds and vitamin C content and, correspondingly, the antioxidant capacity (AC) of blueberries. Significant differences were recorded between the responses of ’Duke’ and ‘Draper’ cultivars to treatments and between the years. In both years, the application of GB (2 kg ha^−1^) generally resulted in a significant increase in the total phenolic content (TPC) of ‘Duke’ blueberries by 22–33% compared with the control. Furthermore, regarding cv. ‘Draper’, the higher dose of this biostimulant (4 kg ha^−1^ GB) contributed to a 16–29% increase in total anthocyanins and individual polyphenols. Accordingly, ‘Draper’ blueberries subjected to this treatment generally had 21–29% higher AC measured by the ABTS^•+^ and CUPRAC methods in 2022. However, both GB doses and the combined application of 2 L ha^−1^ EM + 2 kg ha^−1^ GB reduced vitamin C concentrations in ‘Duke’ blueberries by 10–30% during both years. Conversely, in 2023, both GB doses increased vitamin C content by 40–80% in fruits of the cv. ‘Draper’. In addition, the application of 4 kg ha^−1^ GB and the combination of 4 L ha^−1^ EM + 4 kg ha^−1^ GB increased the AC in blueberries of the cv. ‘Duke’ by 7–12% as measured via the CUPRAC assay in both years. The treatment with 4 L ha^−1^ EM in 2022 increased by 14–21% total anthocyanins, individual polyphenols, and AC according to ABTS^•+^ and CUPRAC methods in ‘Draper’ blueberries. It also increased TPC in ‘Duke’ fruits by 21%. In another way, the application of 2 L ha^−1^ EM reduced anthocyanins, individual phenolics, and AC by the CUPRAC assay by 16–20%. Our findings underscore that the effect of biostimulants is influenced by the applied dose, genotype, and climatic conditions of the year. Therefore, dosages can be modulated based on each cultivar’s specific response and environmental conditions. This study showed the potential of EM- and GB-based biostimulants to enhance the nutritional quality and antioxidant capacity of blueberries.

## Figures and Tables

**Table 1:** Average levels of individual polyphenols (μg g^−1^) (Part 1) of fruits from ‘Duke’ and ‘Draper’ blueberry cultivars (Cv) depending on the treatments (T) used in the years 2022 and 2023.

Treatments	Years	Chlorogenic Acid		Delphinidin-3-*O*-galactoside		Delphinidin-3-*O*-glucoside		Delphinidin-3-*O*-arabinoside		Petunidin-3-*O*-galactoside		Malvidin-3-*O*-galactoside	
		‘Duke’	‘Draper’	‘Duke’	‘Draper’	‘Duke’	‘Draper’	‘Duke’	‘Draper’	‘Duke’	‘Draper’	‘Duke’	‘Draper’
**T0**	**2022**	312.92 ± 7.02 ^ab^	305.97 ± 11.38 ^AB^	1059.47 ± 115.62 ^b^	625.71 ± 12.18 ^A^	858.09 ± 99.31 ^b^	513.18 ± 10.08 ^A^	535.06 ± 61.89 ^b^	378.26 ± 8.25 ^A^	626.64 ± 68.75 ^ab^	392.64 ± 8.99 ^AB^	347.77 ± 40.20 ^a^	247.45 ± 8.44 ^A^
	**2023**	503.61 ± 5.82 ^a^	547.27 ± 12.39 ^A^	975.48 ± 29.61 ^b^	702.77 ± 25.78 ^A^	783.27 ± 11.44 ^c^	599.50 ± 17.43 ^A^	472.41 ± 22.41 ^b^	341.57 ± 8.78 ^ABC^	438.03 ± 22.69 ^bc^	397.31 ± 16.55 ^AB^	341.83 ± 9.16 ^bc^	235.31 ± 9.63 ^A^
**T1**	**2022**	356.32 ± 7.29 ^bc^	376.13 ± 41.08 ^BC^	889.01 ± 2.23 ^ab^	737.12 ± 46.81 ^ABC^	744.40 ± 0.80 ^ab^	610.48 ± 39.47 ^AB^	466.80 ± 1.77 ^ab^	452.35 ± 25.07 ^BC^	609.36 ± 15.60 ^ab^	508.63 ± 28.68 ^B^	311.82 ± 3.03 ^a^	285.87 ± 10.62 ^AB^
	**2023**	621.34 ± 16.37 ^b^	519.59 ± 20.08 ^A^	858.86 ± 56.40 ^a^	711.13 ± 34.96 ^A^	754.37 ± 56.73 ^c^	594.92 ± 36.06 ^A^	541.17 ± 54.43 ^b^	310.60 ± 20.67 ^AB^	489.93 ± 36.86 ^cd^	398.49 ± 17.21 ^AB^	338.99 ± 25.37 ^bc^	237.84 ± 22.38 ^A^
**T2**	**2022**	392.79 ± 27.31 ^c^	422.60 ± 62.23 ^C^	821.88 ± 10.34 ^a^	658.44 ± 25.23 ^AB^	686.90 ± 15.05 ^a^	551.66 ± 25.27 ^A^	404.83 ± 14.40 ^a^	389.21 ± 12.90 ^AB^	528.87 ± 7.48 ^a^	461.57 ± 21.36 ^ABC^	283.65 ± 6.03 ^a^	255.53 ± 13.57 ^A^
	**2023**	502.61 ± 61.38 ^a^	567.43 ± 38.82 ^A^	761.91 ± 10.39 ^a^	696.90 ± 86.44 ^A^	676.35 ± 15.58 ^abc^	576.42 ± 83.04 ^A^	473.44 ± 11.58 ^b^	302.10 ± 39.44 ^B^	508.06 ± 5.57 ^cd^	335.00 ± 38.52 ^A^	326.09 ± 13.27 ^bc^	233.59 ± 37.51 ^A^
**T3**	**2022**	369.32 ± 19.71 ^c^	365.17 ± 29.54 ^ABC^	927.79 ± 48.87 ^ab^	809.15 ± 29.54 ^C^	795.28 ± 45.99 ^ab^	681.19 ± 24.39 ^B^	487.04 ± 38.85 ^ab^	481.78 ± 13.68 ^C^	641.47 ± 32.06 ^b^	505.40 ± 31.30 ^C^	339.14 ± 23.47 ^a^	326.26 ± 18.78 ^B^
	**2023**	552.30 ± 24.23 ^ab^	572.05 ± 14.70 ^A^	866.86 ± 64.57 ^ab^	780.92 ± 47.35 ^A^	775.76 ± 46.69 ^c^	695.70 ± 25.98 ^A^	546.43 ± 44.70 ^b^	419.47 ± 17.13 ^C^	570.33 ± 41.16 ^d^	501.40 ± 13.07 ^C^	359.61 ± 12.42 ^c^	270.92 ± 13.85 ^A^
**T4**	**2022**	379.81 ± 17.87 ^c^	333.11 ± 13.05 ^ABC^	919.28 ± 79.90 ^ab^	722.97 ± 93.80 ^AB^	767.90 ± 71.56 ^ab^	599.12 ± 90.69 ^AB^	487.28 ± 40.82 ^ab^	405.39 ± 47.94 ^AB^	589.99 ± 45.26 ^ab^	414.97 ± 56.57 ^AB^	308.34 ± 25.95 ^a^	285.04 ± 28.63 ^AB^
	**2023**	547.03 ± 37.10 ^ab^	565.86 ± 32.05 ^A^	827.04 ± 38.39 ^a^	681.96 ± 55.06 ^A^	690.96 ± 72.44 ^bc^	592.76 ± 61.64 ^A^	441.86 ± 104.35 ^b^	374.09 ± 48.56 ^ABC^	434.37 ± 76.64 ^bc^	438.91 ± 44.54 ^BC^	298.65 ± 30.27 ^b^	244.94 ± 34.23 ^A^
**T5**	**2022**	395.68 ± 28.92 ^c^	320.85 ± 19.08 ^AB^	934.54 ± 39.06 ^ab^	760.98 ± 35.24 ^BC^	772.69 ± 33.34 ^ab^	620.78 ± 23.33 ^AB^	465.58 ± 27.42 ^ab^	454.72 ± 29.06 ^BC^	614.93 ± 15.67 ^ab^	463.65 ± 24.71 ^BC^	303.01 ± 21.81 ^a^	287.15 ± 3.73 ^AB^
	**2023**	514.08 ± 15.97 ^a^	569.99 ± 47.21 ^A^	772.80 ± 22.81 ^a^	686.79 ± 62.30 ^A^	592.51 ± 17.66 ^ab^	608.93 ± 59.99 ^A^	215.55 ± 43.07 ^a^	389.57 ± 24.38 ^BC^	355.38 ± 23.09 ^ab^	427.22 ± 30.68 ^BC^	227.15 ± 19.52 ^a^	237.80 ± 22.69 ^A^
**T6**	**2022**	291.00 ± 13.85 ^a^	270.85 ± 34.93 ^A^	853.34 ± 61.81 ^a^	651.44 ± 26.92 ^AB^	717.18 ± 48.28 ^ab^	546.88 ± 21.94 ^B^	437.34 ± 28.70 ^ab^	404.83 ± 15.93 ^AB^	599.71 ± 49.04 ^ab^	376.11 ± 20.84 ^A^	297.87 ± 26.15 ^a^	271.48 ± 10.52 ^A^
	**2023**	510.36 ± 28.57 ^a^	506.65 ± 47.57 ^A^	791.19 ± 26.50 ^a^	716.84 ± 43.92 ^A^	569.56 ± 7.34 ^a^	627.69 ± 33.09 ^A^	169.84 ± 2.93 ^a^	357.80 ± 29.26 ^ABC^	291.21 ± 8.98 ^a^	444.47 ± 27.11 ^BC^	209.88 ± 5.24 ^a^	247.40 ± 20.97 ^A^
***p*** **(T)**	**2022**	<0.001		<0.01		<0.05		<0.001		<0.001		<0.001	
	**2023**	<0.05		<0.01		<0.001		<0.001		<0.001		<0.001	
***p*** **(Cv)**	**2022**	>0.05		<0.001		<0.001		<0.001		<0.001		<0.001	
	**2023**	>0.05		<0.001		<0.001		<0.001		<0.05		<0.001	
***p*** **(T × Cv)**	**2022**	<0.05		<0.001		<0.01		<0.01		<0.01		<0.05	
	**2023**	<0.01		<0.01		<0.001		<0.001		<0.001		<0.001	

Values are means ± SD (*n* = 3). Lowercase letters indicate significant differences (*p* < 0.05) between ‘Duke’ treatments and uppercase letters indicate significant differences (*p* < 0.05) between ‘Draper’ treatments by Tukey’s test.

**Table 2:** Average levels of individual polyphenols (μg g^−1^) (Part 2), including the sum of anthocyanins and the sum of polyphenols (mg g^−1^) of fruits from ‘Duke’ and ‘Draper’ blueberry cultivars (Cv) depending on the treatments (T) used in the years 2022 and 2023.

Treatments	Years	Malvidin-3-*O*-arabinoside		Rutin		Hyperoside		Sum of Anthocyanins		Sum of Individual Polyphenols	
		‘Duke’	‘Dr aper’	‘Duke’	‘Draper’	‘Duke’	‘Draper’	‘Duke’	‘Draper’	‘Duke’	‘Draper’
**T0**	**2022**	458.56 ± 50.19 ^b^	358.13 ± 4.24 ^A^	334.50 ± 45.63 ^b^	181.33 ± 4.66 ^AB^	46.69 ± 8.02 ^b^	26.21 ± 0.84 ^A^	3.89 ± 0.43 ^b^	2.52 ± 0.05 ^A^	4.58 ± 0.48 ^b^	3.03 ± 0.05 ^A^
	**2023**	368.81 ± 4.06 ^ab^	366.52 ± 7.77 ^AB^	303.58 ± 13.80 ^cd^	211.04 ± 11.16 ^A^	51.69 ± 2.58 ^c^	35.04 ± 2.51 ^A^	3.38 ± 0.08 ^c^	2.64 ± 0.08 ^AB^	4.24 ± 0.08 ^c^	3.44 ± 0.11 ^AB^
**T1**	**2022**	455.32 ± 4.04 ^b^	465.24 ± 17.07 ^C^	277.36 ± 0.19 ^ab^	191.69 ± 7.89 ^ABC^	40.77 ± 0.26 ^ab^	27.62 ± 1.70 ^A^	3.48 ± 0.03 ^ab^	3.06 ± 0.16 ^BC^	4.15 ± 0.03 ^ab^	3.66 ± 0.21 ^B^
	**2023**	449.66 ± 42.17 ^bc^	386.63 ± 20.45 ^AB^	314.30 ± 24.92^d^	223.24 ± 6.14 ^A^	49.85 ± 5.49 ^bc^	36.38 ± 1.26 ^A^	3.44 ± 0.27 ^c^	2.64 ± 0.15 ^AB^	4.43 ± 0.31 ^c^	3.42 ± 0.17 ^AB^
**T2**	**2022**	381.63 ± 5.83 ^a^	407.18 ± 21.93 ^ABC^	262.13 ± 4.25 ^a^	179.29 ± 11.06 ^A^	35.69 ± 1.77 ^a^	26.62 ± 1.70 ^A^	3.10 ± 0.04 ^a^	2.72 ± 0.12 ^AB^	3.79 ± 0.05 ^a^	3.35 ± 0.17 ^AB^
	**2023**	445.03 ± 7.29 ^bc^	313.27 ± 45.91 ^A^	252.47 ± 2.37 ^ab^	199.74 ± 23.20 ^A^	40.61 ± 0.65 ^ab^	32.99 ± 4.49 ^A^	3.19 ± 0.06 ^c^	2.45 ± 0.33 ^A^	3.99 ± 0.11 ^bc^	3.25 ± 0.39 ^A^
**T3**	**2022**	478.61 ± 30.11 ^b^	448.31 ± 22.55 ^BC^	296.45 ± 13.57 ^ab^	248.35 ± 10.20^D^	40.88 ± 1.98 ^ab^	36.09 ± 1.08 ^B^	3.67 ± 0.22 ^ab^	3.25 ± 0.14 ^C^	4.38 ± 0.25 ^ab^	3.90 ± 0.11 ^B^
	**2023**	489.92 ± 45.05 ^c^	490.00 ± 13.34 ^C^	266.78 ± 20.12 ^abc^	226.21 ± 11.80 ^A^	43.77 ± 5.14 ^abc^	38.56 ± 2.25 ^A^	3.61 ± 0.25 ^c^	3.16 ± 0.08 ^B^	4.47 ± 0.30 ^c^	4.00 ± 0.08 ^B^
**T4**	**2022**	425.98 ± 29.23 ^ab^	386.79 ± 46.08 ^AB^	313.38 ± 24.39 ^ab^	218.98 ± 18.69 ^CD^	42.24 ± 4.59 ^ab^	30.37 ± 3.08 ^A^	3.50 ± 0.29 ^ab^	2.81 ± 0.35 ^ABC^	4.23 ± 0.33 ^ab^	3.40 ± 0.38 ^AB^
	**2023**	356.46 ± 49.79 ^a^	396.14 ± 41.32 ^AB^	283.69 ± 8.41 ^bcd^	203.81 ± 21.38 ^A^	46.19 ± 2.00 ^bc^	33.21 ± 5.63 ^A^	3.05 ± 0.36 ^bc^	2.73 ± 0.28 ^AB^	3.93 ± 0.37 ^bc^	3.53 ± 0.34 ^AB^
**T5**	**2022**	448.53 ± 17.92 ^ab^	407.06 ± 18.41 ^ABC^	302.33 ± 14.05 ^ab^	211.18 ± 11.47 ^BC^	40.71 ± 1.53 ^ab^	29.40 ± 0.54 ^A^	3.54 ± 0.15 ^ab^	2.99 ± 0.13 ^ABC^	4.28 ± 0.16 ^ab^	3.56 ± 0.15 ^AB^
	**2023**	416.40 ± 7.02 ^abc^	379.47 ± 27.45 ^AB^	255.46 ± 11.93 ^ab^	201.86 ± 15.97 ^A^	40.33 ± 1.21 ^ab^	32.22 ± 4.24 ^A^	2.58 ± 0.12 ^ab^	2.73 ± 0.22 ^AB^	3.39 ± 0.12 ^ab^	3.53 ± 0.29 ^AB^
**T6**	**2022**	430.81 ± 26.91 ^ab^	359.18 ± 22.07 ^A^	265.45 ± 20.06 ^a^	181.23 ± 6.87 ^AB^	35.01 ± 2.71 ^a^	25.80 ± 1.56 ^A^	3.34 ± 0.24 ^ab^	2.61 ± 0.11 ^AB^	3.93 ± 0.27 ^ab^	3.09 ± 0.14 ^AB^
	**2023**	403.93 ± 7.32 ^ab^	409.25 ± 31.93 ^BC^	226.16 ± 21.84 ^a^	199.82 ± 11.49 ^A^	35.13 ± 3.97 ^a^	32.67 ± 1.16 ^A^	2.44 ± 0.04 ^a^	2.80 ± 0.18 ^AB^	3.21 ± 0.09 ^a^	3.54 ± 0.22 ^AB^
***p*** **(T)**	**2022**	<0.001		<0.001		<0.001		<0.01		<0.01	
	**2023**	<0.001		<0.001		<0.001		<0.001		<0.001	
***p*** **(Cv)**	**2022**	<0.001		<0.001		<0.001		<0.001		<0.001	
	**2023**	<0.01		<0.001		<0.001		<0.001		<0.001	
***p*** **(T × Cv)**	**2022**	<0.01		<0.01		<0.01		<0.01		<0.01	
	**2023**	<0.001		<0.01		<0.05		<0.001		<0.001	

Values are means ± SD (*n* = 3). Lowercase letters indicate significant differences (*p* < 0.05) between ‘Duke’ treatments and uppercase letters indicate significant differences (*p* < 0.05) between ‘Draper’ treatments by Tukey’s test.

**Table 3:** Antioxidant capacity (AC) of fruits from ‘Duke’ and ‘Draper’ blueberry cultivars (Cv) depending on the treatments (T) used in the years 2022 and 2023.

Treatments	Years	ABTS^•+^ (μmol L^−1^ TE g^−1^)		CUPRAC (μmol L^−1^ TE g^−1^)		DPPH^•^ (μmol L^−1^ TE g^−1^)		FRAP (μmol L^−1^ FeSO_4_ g^−1^)	
		‘Duke’	‘Draper’	‘Duke’	‘Draper’	‘Duke’	‘Draper’	‘Duke’	‘Draper’
**T0**	**2022**	149.89 ± 13.12 ^ab^	86.03 ± 3.40 ^A^	119.48 ± 5.02 ^b^	88.49 ± 0.35 ^A^	82.60 ± 9.29 ^a^	77.21 ± 0.19 ^B^	178.69 ± 0.11 ^a^	151.25 ± 1.36 ^AB^
	**2023**	176.50 ± 5.38 ^abc^	169.53 ± 1.43 ^AB^	72.60 ± 4.13 ^b^	60.57 ± 10.75 ^A^	118.47 ± 2.61 ^a^	95.57 ± 2.86 ^A^	96.01 ± 2.83 ^c^	81.19 ± 3.57 ^A^
**T1**	**2022**	154.24 ± 0.38 ^b^	100.90 ± 1.83 ^BC^	118.27 ± 2.10 ^b^	101.27 ± 2.77 ^BC^	91.86 ± 3.42 ^a^	66.12 ± 8.85 ^AB^	187.49 ± 0.54 ^abc^	145.64 ± 3.94 ^A^
	**2023**	196.32 ± 0.78 ^d^	176.16 ± 3.77 ^B^	72.57 ± 0.16 ^b^	64.89 ± 3.77 ^A^	146.65 ± 5.83 ^b^	104.18 ± 5.04 ^A^	91.47 ± 1.69 ^abc^	81.53 ± 1.58 ^A^
**T2**	**2022**	128.10 ± 4.94 ^a^	91.36 ± 0.75 ^AB^	100.88 ± 2.20 ^a^	100.70 ± 4.05 ^BC^	97.09 ± 3.14 ^a^	71.44 ± 6.63 ^AB^	180.64 ± 1.61 ^ab^	154.43 ± 4.58 ^AB^
	**2023**	176.18 ± 8.87 ^ab^	172.40 ± 4.65 ^AB^	72.92 ± 2.49 ^b^	69.99 ± 0.86 ^A^	133.90 ± 6.13 ^ab^	102.42 ± 11.57 ^A^	95.05 ± 1.80 ^bc^	83.95 ± 2.68 ^A^
**T3**	**2022**	142.35 ± 8.15 ^ab^	110.87 ± 1.62 ^CD^	128.39 ± 4.35 ^c^	107.24 ± 6.45 ^C^	97.11 ± 0.70 ^a^	73.90 ± 5.54 ^AB^	183.69 ± 3.17 ^abc^	159.03 ± 0.24 ^B^
	**2023**	189.58 ± 0.16 ^bcd^	176.85 ± 7.42 ^B^	81.01 ± 0.43 ^c^	66.82 ± 7.00 ^A^	119.55 ± 18.15 ^a^	109.91 ± 2.88 ^A^	92.34 ± 1.13 ^abc^	85.49 ± 1.83 ^A^
**T4**	**2022**	134.85 ± 11.02 ^ab^	117.69 ± 7.86 ^D^	123.92 ± 1.16 ^bc^	99.33 ± 4.45 ^BC^	95.71 ± 4.58 ^a^	62.90 ± 2.14 ^AB^	181.95 ± 5.68 ^ab^	147.00 ± 6.43 ^A^
	**2023**	190.03 ± 5.51 ^cd^	170.83 ± 8.80 ^AB^	62.81 ± 0.16 ^a^	57.50 ± 4.66 ^A^	108.73 ± 9.16 ^a^	96.21 ± 6.24 ^A^	87.10 ± 1.74 ^a^	84.71 ± 4.67 ^A^
**T5**	**2022**	143.80 ± 3.61 ^ab^	107.51 ± 3.38 ^CD^	130.29 ± 1.76 ^c^	101.08 ± 2.13 ^BC^	98.13 ± 3.48 ^a^	60.20 ± 5.34 ^A^	194.76 ± 4.29 ^c^	147.07 ± 4.04 ^A^
	**2023**	167.15 ± 4.98 ^a^	157.08 ± 4.77 ^A^	80.42 ± 0.71 ^c^	55.61 ± 13.36 ^A^	109.48 ± 4.44 ^a^	104.43 ± 6.67 ^A^	90.29 ± 2.12 ^ab^	93.37 ± 1.95 ^B^
**T6**	**2022**	137.02 ± 9.75 ^ab^	91.16 ± 3.00 ^AB^	129.54 ± 3.50 ^c^	92.59 ± 0.71 ^AB^	81.89 ± 16.96 ^a^	75.06 ± 2.42 ^B^	190.64 ± 7.16 ^bc^	158.60 ± 1.07 ^B^
	**2023**	182.30 ± 2.19 ^bc^	169.35 ± 4.92 ^AB^	75.44 ± 5.16 ^bc^	72.41 ± 2.88 ^A^	108.83 ± 8.07 ^a^	106.21 ± 2.03 ^A^	88.74 ± 1.42 ^a^	84.33 ± 0.58 ^A^
***p*** **(T)**	**2022**	<0.001		<0.001		>0.05		<0.001	
	**2023**	<0.001		<0.01		<0.001		<0.01	
***p*** **(Cv)**	**2022**	<0.001		<0.001		<0.001		<0.001	
	**2023**	<0.001		<0.001		<0.001		<0.001	
***p*** **(T × Cv)**	**2022**	<0.001		<0.001		<0.01		<0.001	
	**2023**	>0.05		<0.05		<0.001		<0.001	

Values are means ± SD (*n* = 3). Lowercase letters indicate significant differences (*p* < 0.05) between ‘Duke’ treatments and uppercase letters indicate significant differences (*p* < 0.05) between ‘Draper’ treatments by Tukey’s test.

## Data Availability

The original contributions presented in this study are included in the article/supplementary material. Further inquiries can be directed to the corresponding author.

## Supplementary Materials

The following supporting information can be downloaded at https://www.mdpi.com/article/10.3390/plants15010092/s1; Figure S1: Representative HPLC chromatogram of polyphenols in blueberry extract recorded at 280, 320, 370, and 520 nm.

## Appendix A

**Table A1:** Statistical effect of treatment (T), cultivar (Cv), year (Y), and their interactions on the analyzed blueberry parameters.

Parameters	*p* (T)	*p* (Cv)	*p* (Y)	*p* (T × Cv)	*p* (T × Y)	*p* (C × Y)	*p* (T × Cv × Y)
Total Phenolic Compounds	<0.001	<0.001	>0.05	<0.001	>0.05	<0.001	<0.001
Flavonoids	<0.001	<0.001	<0.001	>0.05	<0.05	<0.001	<0.05
*Ortho*-diphenols	<0.01	<0.01	<0.001	>0.05	>0.05	>0.05	>0.05
Sum of Anthocyanins	<0.001	<0.001	<0.001	<0.001	<0.05	<0.01	<0.001
Sum of Individual Polyphenols	<0.001	<0.001	>0.05	<0.001	>0.05	<0.01	<0.001
Vitamin C	<0.001	<0.001	<0.001	<0.001	<0.001	>0.05	<0.001
ABTS^•+^	<0.001	<0.001	<0.001	<0.001	<0.001	<0.001	<0.001
CUPRAC	<0.001	<0.001	<0.001	<0.001	<0.001	<0.001	<0.001
DPPH^•^	<0.001	<0.001	<0.001	<0.001	<0.01	>0.05	<0.001
FRAP	<0.001	<0.001	<0.001	<0.01	<0.01	<0.001	<0.001
