# Supplementary material for: Effect of Foliar Biostimulant Application on Bioactive Compounds and Antioxidant Capacity in Blueberry (Vaccinium corymbosum L.)
Source: Plants (Basel). 2025 Dec 27;15(1):92. doi: 10.3390/plants15010092 (PMC12787387; doi:10.3390/plants15010092)
Supplement: Supplementary file 1 [file plants-15-00092-s001.zip › plants-3908609-supplementary.pdf]

# Supplementary Materials

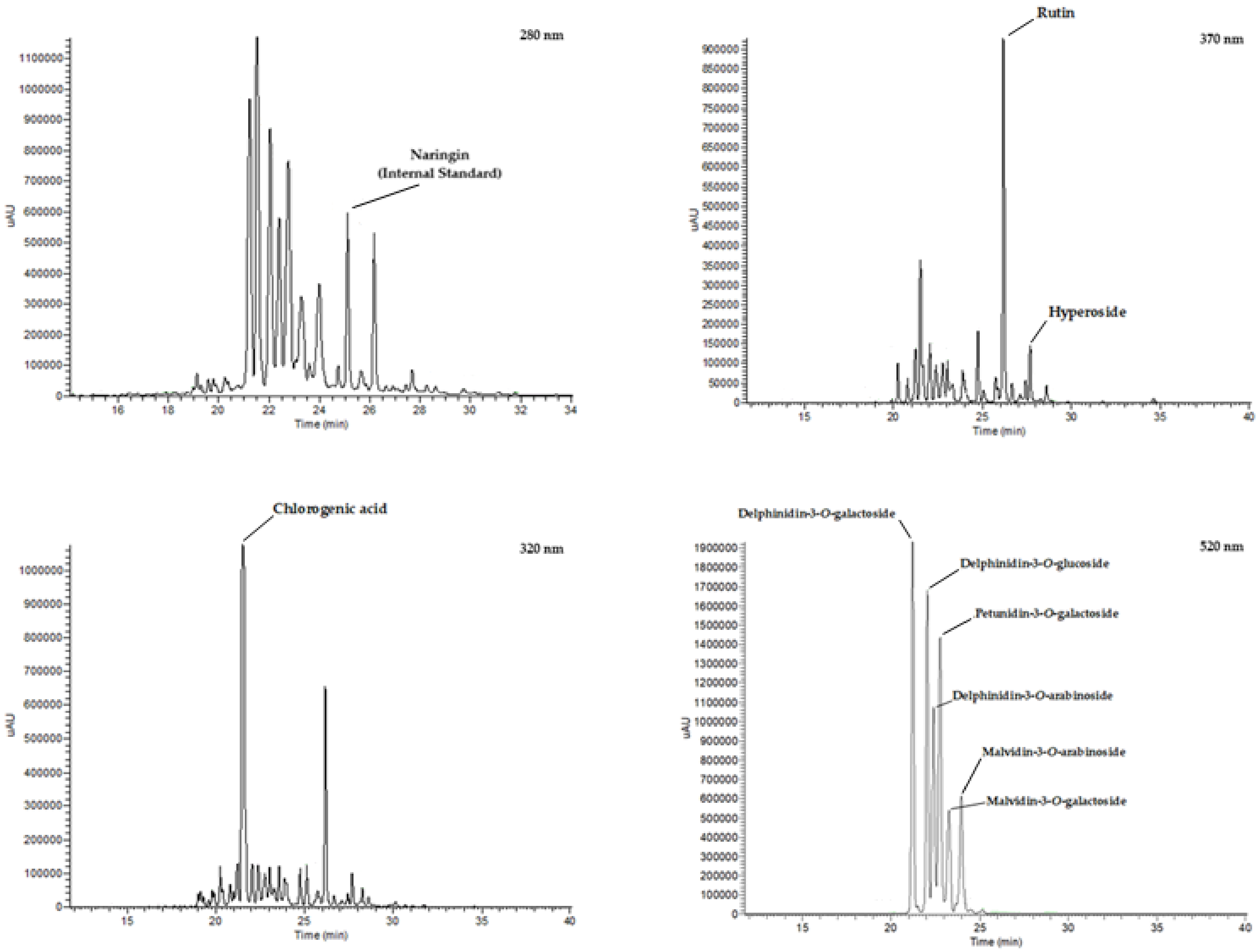


**Figure S1:** Representative HPLC chromatogram of polyphenols in blueberry extract recorded at 280, 320, 370, and 520 nm.
